# Evolution of brain injury and neurological dysfunction after cardiac arrest in the rat – A multimodal and comprehensive model

**DOI:** 10.1177/0271678X241255599

**Published:** 2024-05-21

**Authors:** Carlo Perego, Francesca Fumagalli, Francesca Motta, Marianna Cerrato, Edoardo Micotti, Davide Olivari, Daria De Giorgio, Giulia Merigo, Angelo Di Clemente, Alessandra Mandelli, Gianluigi Forloni, Luigi Cervo, Roberto Furlan, Roberto Latini, Robert W Neumar, Giuseppe Ristagno

**Affiliations:** 1 Department of Acute Brain and Cardiovascular Injury, Istituto di Ricerche Farmacologiche Mario Negri IRCCS, Milan, Italy; 2 Department of Neuroscience Istituto di Ricerche Farmacologiche Mario Negri IRCCS, Milan, Italy; 3 Department of Anesthesiology, Intensive Care and Emergency, Fondazione IRCCS Ca' Granda Ospedale Maggiore Policlinico, Milan, Italy; 4 Clinical Neuroimmunology Unit, Division of Neuroscience, Institute of Experimental Neurology - INSpe San Raffaele Scientific Institute, Milan, Italy; 5 Department of Emergency Medicine and Max Harry Weil Institute for Critical Care Research and Innovation, University of Michigan, Ann Arbor, Michigan, USA; 6 Department of Pathophysiology and Transplantation, University of Milan, Milan, Italy

**Keywords:** Cardiac arrest, neuroimaging, neurodegeneration, inflammation, NfL

## Abstract

Cardiac arrest (CA) is one of the leading causes of death worldwide. Due to hypoxic ischemic brain injury, CA survivors may experience variable degrees of neurological dysfunction. This study, for the first time, describes the progression of CA-induced neuropathology in the rat. CA rats displayed neurological and exploratory deficits. Brain MRI revealed cortical and striatal edema at 3 days (d), white matter (WM) damage in corpus callosum (CC), external capsule (EC), internal capsule (IC) at d7 and d14. At d3 a brain edema significantly correlated with neurological score. Parallel neuropathological studies showed neurodegeneration, reduced neuronal density in CA1 and hilus of hippocampus at d7 and d14, with cells dying at d3 in hilus. Microgliosis increased in cortex (Cx), caudate putamen (Cpu), CA1, CC, and EC up to d14. Astrogliosis increased earlier (d3 to d7) in Cx, Cpu, CC and EC compared to CA1 (d7 to d14). Plasma levels of neurofilament light (NfL) increased at d3 and remained elevated up to d14. NfL levels at d7 correlated with WM damage. The study shows the consequences up to 14d after CA in rats, introducing clinically relevant parameters such as advanced neuroimaging and blood biomarker useful to test therapeutic interventions in this model.

## Introduction

Sudden cardiac arrest (CA) is the most common cause of death worldwide.^1^ Even though high quality cardiopulmonary resuscitation (CPR) and post-resuscitation care have improved survival,^2^ CA outcome still remains poor, with less than 10% of patients treated for out-of-hospital CA surviving to hospital discharge.^1^ Indeed, brain injury accounts for the majority of deaths in patients admitted to intensive care unit after resuscitation, and is ultimately the main determinant of poor functional outcome, ranging from minor cognitive deficit to persistent coma,^3^ with no definitively proven specific treatments.^4,5^

After CA, the whole body is exposed to an ischemic injury for a variable amount of time, including both no- and low-flow intervals, followed by an abrupt reperfusion when return of spontaneous circulation (ROSC) is achieved. The pathophysiological events of post-CA brain inury have not been entirely revealed yet. Identified damaging pathways include excitotoxicity, arrest of aerobic metabolism, disruption of calcium homeostasis, oxidative stress, release of pro-apoptotic proteins, neuroinflammation, mitochondrial dysfunction, and impairment of cerebrovascular autoregulation.^6^ Our understanding of the processes linking the acute phase after CA to the consequent neurodegenerative pathology is still incomplete, partly because of the paucity of relevant pre-clinical models of CA survival.

In this study, the evolution of brain injury and behavioral outcomes following CA have been longitudinally analyzed in an established rat model in a standardized manner. Thus, a comprehensive multimodal approach, derived from the clinical scenario, has been employed to describe the progression of the pathological events in different brain areas up to 14 days after CA, in relationship with the concurrent functional recovery. Using *in vivo* quantitative magnetic resonance imaging (MRI) with parallel neuropathological analyses and blood biomarker, the onset and progression of the brain injury after CA have been described in detail.

## Methods

This article adheres to the American Heart Association Journals implementation of the Transparency and Openness Promotion Guidelines. The detailed description of procedures can be found in the Data Supplement file. The datasets generated and analyzed during the current study can be found at https://zenodo.org/records/11186427.

## Animals

Male Sprague-Dawley ex-breeder rats were used (weighting 479 ± 15 g, Envigo RMS srl, Italy). Animals were housed in certified specific pathogen-free vivaria. The IRFMN adheres to the principles set out in the following laws, regulations, and policies governing the care and use of laboratory animals: Italian Governing Law (D.lgs 26/2014; Authorisation n.19/2008-A issued March 6, 2008 by Ministry of Health); Mario Negri Institutional Regulations and Policies providing internal authorisation for persons conducting animal experiments (Quality Management System Certificate – UNI EN ISO 9001:2015 – Reg. N° 6121); the NIH Guide for the Care and Use of Laboratory Animals (2011 edition) and EU directives and guidelines (EEC Council Directive 2010/63/UE). All procedures fulfil the criteria of the ARRIVE (Animal Research: Reporting of In Vivo Experiments) guidelines (https://www.nc3rs.org.uk/arrive-guidelines) check list provided in supplemental materials.

## Animal preparation

Before surgery, animals were fasted overnight, with free access to water. They were anesthetized by intraperitoneal (IP) injection of thiopental l (50 mg/kg). Additional doses of thiopental (10 mg/kg) were given at intervals of approximately 40 minutes or when required to maintain anaesthesia. Total doses (mg/kg) of administered thiopental in CA/CPR groups were (mean±SD): 66,9 ± 8,5 for d3; 71,0 ± 7,4 for d7; 68,2 ± 6,4 for d14, with no statistical difference among groups. Ampicillin (50 mg/kg) was injected intramuscularly (IM) as prophylaxis after induction of anaesthesia. Animals were then instrumented for hemodynamic measurements and induction of cardiac arrest (CA) according to our established model of electrically induced CA and CPR. Briefly, the trachea was orally intubated with a 14-gauge cannula. A PE-50 catheter was advanced into the descending aorta from the left femoral artery for measurements of arterial pressure (systolic, median and diastolic arterial pressure: SAP, MAP and DAP) and blood sampling. Through the left external jugular vein, another PE-50 catheter was advanced into the right atrium for measurement of right atrial pressure (RAP) and for the administration of epinephrine. Aortic and right atrial pressures were measured with reference to the mid-chest with conventional external pressure transducers. A 3-Fr PE catheter was advanced through the right external jugular vein into the right atrium. A pre-curved guide wire supplied with the catheter was then advanced through the catheter into the right ventricle for inducing CA. All catheters were flushed intermittently with saline containing 2.5 IU/mL of bovine heparin. A conventional lead II electrocardiogram (ECG, DataQ, Akron, OH) was continuously monitored. Temperature was monitored with the aid of a rectal probe (Physitemp instrument INC. Clifton, NJ) and maintained at 37 ± 0,5°C. The same anaesthetic and surgical procedures were performed for the sham-operated rats excluding cardiac arrest and cardiopulmonary resuscitation.

## Cardiac arrest (CA) and cardiopulmonary resuscitation (CPR) procedures

Rats (n = 43) were randomized^7^ to CA/CPR or sham surgery, as previously described.^8^ Ventricular fibrillation (VF) was electrically induced with 60-Hz current (4 mA) delivered to the right ventricular endocardium. The current flow was maintained for 3 min to prevent spontaneous defibrillation. Animals were subjected 8 minutes of untreated VF following by precordial compression (PC) with a pneumatically driven mechanical chest compressor. The PC depth was adjusted to ensure *coronary perfusion pressure* (CPP) at least of 20–25 mmHg. The PC rate was 200/min with equal compression-decompression. From the start of PC, animals were mechanically ventilated at a frequency of 50/min with tidal volume 0.6 mL/100g and FiO_2_ 1.0. A single dose of epinephrine (0.02 mg/kg) was injected into the right atrium 2 min after the start of PC. After 8 minutes of CPR, resuscitation was attempted with up to three 2-joule (J) defibrillations (CodeMaster XL, Philips Heartstream). ROSC was defined as the return of supraventricular rhythm with MAP >50 mmHg for at least 5 minutes. If ROSC did not occur, two more 1 minute cycles CPR were done with counter-shock. After ROSC, mechanical ventilation was maintained at FiO_2_ 1 for 1 h post-resuscitation, then continued with FiO_2_ 0.21. Blood samples were serially collected from the femoral artery cannula 15 minutes before CA (BL), and 1 and 3 hours after ROSC. Ampicillin (50 mg/kg) was injected intramuscularly as prophylaxis after induction of anaesthesia. Three hours after ROSC, animals returned to their cages. Ampicillin (50 mg/kg intramuscular) and buprenorphine (0.16 mg/kg) were given to prevent infection and pain. CA/CPR was performed on 32 rats. Four CA/CPR rats were excluded because of spontaneous ROSC. Post-ROSC mortality at 72 h was 38%. Sham-operated rats (n = 8) received the identical anesthesia and surgical procedure without CA induction. Since the rapid decrease in body temperature in the rat during the cardiac arrest phase might result in neuroprotective effects, aiming to maximize brain injury after CA/CPR, we maintained the body temperature not lower than 35.5 °C during CA/CPR.

## Inclusion and exclusion criteria

Four CA/CPR rats were excluded because of spontaneous ROSC during the 8 minutes of untreated VF. DTI values of one CA rat at d7 and ADC maps of one CA rat at d3 (and then respective NDS value) were excluded due to image artefact. No extra animals were added to fill the group objective.

## Behavioural tests

Neurological dysfunction was assessed by Neurological deficit scores (NDS),^9^ tape removal test (TRT).^10^ TRT measured the time until the animal removed adhesive tapes (adhesive tape size was 10-mm by 12-mm) from both their fore paws. The test was truncated at 180 sec.^10^

Rats spontaneous locomotor activity was evaluated during light and dark phase of day for 3 days before and 12 days after the cardiac arrest or sham surgery (light phase between 7 AM and 7 PM; dark phase between 7 PM and 7 AM). Rats were kept in individual transparent cages (42 × 28 × 21 cm, length × width ×height) with sawdust bedding. Each cage was placed between metal frames (54 × 50 × 37 cm) holding two sets of parallel photo beams, crossing the cage 3 cm above the floor (Multiple Activity Cage, Ugo Basile, Varese, Italy). Further details in supplemental material. To see whether sham and CA rats exhibited changes in exploratory and motor activity, animals were tested in an open field 13d after the CA or sham surgery. (as detailed in supplemental material).

## Brain magnetic resonance imaging acquisitions

**Figure 1.**
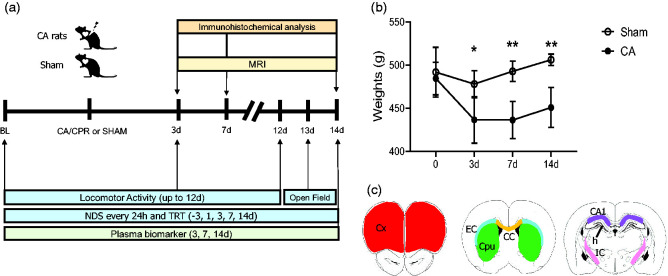
(a) Experimental design. Baseline values (BL) before surgery were obtained at day (d) −1 for neurological deficit score (NDS), tape removal test (TRT) and from −3d for locomotor activity. NDS, TRT and MRI analysis were assessed at multiple intervals up to 14d (up to 12d for locomotor activity and at 13d open field) after surgery (CA/CPR or sham). Additional sub-groups of animals were sacrificed at 3d and 7d after CA/CPR for immunohistochemical analysis and plasma biomarkers. (b) Body weights of sham and CA rats after surgery. Two-way ANOVA for repeated measurements. p = 0.0012, *p < 0.05, **p < 0.01 CA vs sham, Šídák's multiple comparisons test. Data are mean ± SD, n = 8 and (c) regions of interest (ROIs) for histopathological analysis in the brain of sham or CA rats. Drawings represent the ROI selected for quantification: cortex (Cx, red), Caudate putamen (Cpu, green), external capsula (EC, light blu) and corpus callosum (CC, yellow). CA1 (violet), hilus (h) and internal capsule (IC, pink).

Brain imaging was done on a 7 T small-bore animal scanner (BioSpec®; Bruker, Ettlingen, Germany) running ParaVision 6.01 (Bruker, Ettlingen, Germany). To characterize post-CA brain injury, diffusion-weighted imaging (DWI) and diffusion tensor imaging (DTI) sequences were acquired for quantification of cerebral edema and white matter damage respectively at 3, 7 and 14 days after CA. Anaesthetised rats (isoflurane 1.5–2 vol% in an N2O/O2 (70%/30%) were positioned in the magnet at the timepoints indicated in Figure 1. Respiratory frequency was monitored throughout the experiment and body temperature was maintained at 37 °C with a heating pad.

The severity of white matter damage was quantified by comparing the values of fractional anisotropy (FA) and diffusivity (radial (RA), axial (AD) obtained from DTI sequences. The apparent diffusion coefficient (ADC) was used as a quantitative measurement of water diffusion changes in the brain. Intracerebral cytotoxic edema reduces water diffusivity and this corresponded to a reduction in ADC values.

Diffusion weighted echo-planar images (TR/TE = 7000/31.2 ms, slice thickness = 0.6 mm, in-plane resolution = 0.156 × 0.156 mm^2^) covering the brain from olfactory bulbs to the beginning of cerebellum, were adopted to obtain the apparent diffusion coefficient (ADC)-maps. Diffusion-encoding was applied in 3 orthogonal directions with b values of 700 s/mm^2^, respectively. ADC-maps were calculated on a pixel-by-pixel basis using the model function: ln(S(b)/S0) = −b⋅ADC, where S(b) is the measured signal intensity at a specific b value (b) and S0 the signal intensity in the absence of a diffusion gradient (b = 0). The different ADC values per region were calculated. ADC maps in sham operated rats revealed a mean grey matter value of 0.62 ± 0.04. In line with clinical studies, cytotoxic edema was revealed in images from CA rats when threshold was performed with a lower cut-off value of 0.52 µm^2^/msec. An expert operator blind to experimental condition extracted the ADC values in thresholded map using freely available ITK-SNAP software. Brain areas from sham and CA rats were selected manually by a trained expert following the rat brain atlas.^11^ The average ADC value within each region was computed for each animal. Group average ADC values were reported for each region and the mean of the analysed regions was reported as total.

Diffusion tensor imaging (DTI): echo-planar imaging sequences were acquired (TR/TE = 6000/21.5 ms, slice thickness = 0.6 mm, in-plane resolution = 0.178 ×0.178 mm^2^) with the same geometry as DWI images. Diffusion encoding b factors of 700 s/mm^2^ were applied along 19 isotropic directions and two B0 unweighted images for each repetition. The diffusion tensor was computed using FSL software. A group mean full tensor template was first created using a population-based DTI atlas construction algorithm that adopts a tensor-based registration procedure embedded in the DTI-TK software library. The average template was then resampled to an in-plane resolution of 100 ×100 μm^2^ and slice thickness 0.2 mm, and skeletonized. FA images of all subjects were normalized to the mean template with a diffeomorphic transformation and the transformations were applied to all the DTI diffusivity metrics (radial, axial), which were warped to the mean skeleton for region of interest (ROI)-based analysis.

Qualitative MRI variables used from DTI sequences were axial (AD), radial diffusivity (RD) and fractional anisotropy (FA). Axial diffusivity represents the rate of water diffusion along the principal axis parallel to the main vector of the white matter fiber, while radial diffusivity indicates the rate of diffusion perpendicular to the main vector. These values reflect respectively, axonal degeneration and myelin loss. Fractional anisotropy provides instead a cumulative direction of water diffusion, suggesting that lower value of FA indicates a loss of directionality of water molecule diffusion in the white matter tracts representing microstructural damage of the white matter fibers. ROIs were the corpus callosum (CC), the external capsule (EC), the internal capsule (IC) and were selected manually on the reference template by a trained expert following the rat brain atlas.^11^ Further details in the supplemental material.

## Tissue processing and plasma assay

At 3,7 and 14d after sham or CA surgery, rats were euthanized with an intraperitoneal injection of pentobarbital sodium (150 mg/kg). Blood samples were collected from the descending aorta artery in 3 K‐EDTA tubes. Rats were then perfused via the ascending aorta with cold phosphate-buffered saline (PBS), 0.1 mol/l, pH7.4, followed by chilled paraformaldehyde (4%) in PBS. After decapitation the brains were carefully removed from the skull and post-fixed for 6 h at 4 °C, and then transferred to 30% sucrose in 0.1 mol/l PBS for 24 h until equilibration. The brains were frozen by immersion in isopentane at −45°C for 3 minutes before being sealed into vials and stored at −80°C until use. Coronal brain cryosections 20 μm thick were cut serially at −20°C (CM1850UV Leica Biosystems, Germany) and stored at 4 °C in a solution of glycerol:PBS (1:1). Brain slices from sham and CA rats were matched for anteroposterior level. Neuronal cell loss was evaluated on cresyl violet stained sections. Methodological details on plasma assay can be found in the supplemental material.

## Immunohistochemistry

Immunohistochemistry was done on 20 μm brain coronal sections incubated overnight at 4 °C with anti-Iba1 (1:200; Wako, Neuss, Germany) to detect microglia macrophage activation and with primary monoclonal antibody mouse anti-mouse glial fibrillary acid protein (GFAP, 1:2000, Millipore, Billerica, MA, USA). Biotinylated secondary antibodies (1:200, Vector Laboratories, CA, USA) were used. Positive cells were stained by reaction with 3,3 diaminobenzidine tetrahydrochloride (DAB, Vector laboratories, CA, USA). Negative control studies, without the primary antibody, were performed in parallel. Methodological details on Fluoro Jade labelling and TUNEL staining can be found in the supplemental material.

## Slice selection and image acquisition

Three brain coronal sections per rat at +4.68, +0.60 and −3.48 mm from bregma,^11^ were used to quantify neuronal cells and immunostainings. Slices from sham and CA rats were matched for anteroposterior level. The entire brain sections were acquired at 20X with a pixel size of 0.346 µm (10× magnification, with a pixel size of 0.694 μm for F-J and TUNEL) by an Olympus BX-61 Virtual Stage microscope equipped with motorized platform (Olympus, Hamburg, Germany) and digitized.^12^ Acquisition was done over 10 μm thick stacks, with a step size of 2 μm. The different focal planes were merged into a single stack by mean intensity projection to ensure consistent focus throughout the sample.

## Definition of regions of interest (ROI) and image quantification

Images were analyzed using Fiji software (https://fiji.sc/). The regions of interest overlap the recorded MRI-ADC hypointense regions and were defined as depicted in in Figure 1. They included the primary and secondary motor cortex, the medial ventral and lateral orbital cortex, the frontal cortex (AP 4.68); the caudate putamen (Cpu) (AP 0.60); the CA1, CA2, CA3 field of hippocampus, the dentate girus and hilus (AP-3.48) and were selected manually by a trained expert following Paxinos atlas.^11^

Neuronal cell loss was expressed as density of cells/mm^2^ within each side. The cell count of healthy neurons (and immunostaining quantifications) was performed on the entire ROI of right and left hemispheres as depicted Figure 1 as described previously and reported in supplemental materials.^12^

Methodological details on plasma assay can be found in the supplemental material.

## Statistical analysis

Data are presented as box and whiskers with line at mean and min-to-max. For plasma cerebral biomarker data are expressed as medians with interquartile range. Group size was defined having the Neurological Deficit Score (NDS) at 72 hours after CA/CPR as primary outcome. In line with our previous data^8^ to detect effect of CA/CPR (power = 0.8; alpha = 0.05, 1-sided) the group size resulted in n = 8 as number of rats to be used. Since the mean survival rate at 72 hours after CA was 62%, CA/CPR was performed in 13 rats for longitudinal studies. As secondary outcome, a previously unexplored histopathological assessment, 11 rats (CA/CPR performed in 18 rats) were used for additional sub-groups of post-CA timepoints (3d, n = 5; 7d n = 6). The lower number of rats was justified by the drop-out rate of our model. Standard software packages GraphPad Prism (GraphPad Software, Inc, San Diego, CA, version 7.0) were used. Differences between groups over time (group-by-time interaction) for continuous variables were tested using two-way analysis of variance for repeated measurements (time points Groups were compared using One-way ANOVA or Two-way ANOVA followed by an appropriate *post hoc* test. Datasets with unequal variances were analyzed using Brown-Forsithe and Welch corrected ANOVA in order to satisfy the assumptions required by the models. Two-tail p-values lower than 0.05 were considered statistically significant. The parametric or non-parametric test was selected after a Shapiro-Wilk normality test to assess whether groups met normal distribution.

## Results

Two groups of animals (CA/CPR and sham rats) were longitudinally evaluated for behavioral, MRI analysis post-CA and then for histological analysis and plasma biomarkers at 14d. Additional sub-groups of animals were evaluated for behavioral, MRI and sacrificed at 3d and 7d after CA for immunohistochemical analysis and plasma biomarker (Figure 1(a)).

## Body weight

As expected, CA induced a significant loss of body weight compared with sham rats at all time points considered (Figure 1(b)).

## CA induces functional deficits

**Figure 2.**
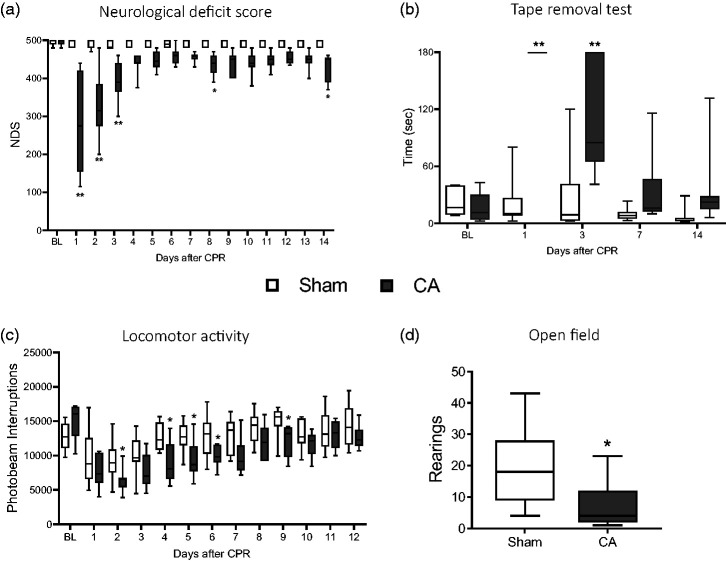
Behavioral deficits. Sensorimotor deficits (NDS, a) and (TRT, b). Analysis of locomotor activity in the dark phase up to 12d in CA and sham rats (c). Number of rearings in Open Fields test at 13d after surgery (CA/CPR or sham). Two-way ANOVA for repeated measurements followed by Sidak’s multiple comparisons test (a, b). Uncorrected Fisher’s LSD (c) and two tailed Student-t test (d). *p < 0.05, **p < 0.01 vs sham; n = 8.

No difference in neurological deficit score (NDS) was observed between groups at baseline (BL, Figure 2(a)). The score indicated significant sensorimotor deficits in CA compared to sham rats up to 3 days (d) after CPR (Figure 2(a)). CA rats showed an initial poor score of 300 out of 500, with subsequent improvement over the next ten days up to a score of 450, with a persistent deficit compared to sham at d14 (Figure 2(a)). Sham rats had NDS in the range of 495 to 500.

At baseline (BL), all rats removed the tape quickly, i.e. <30 sec, with no difference between groups (Figure 2(b)). The tape removal test (TRT) showed sensorimotor integration deficit in CA/CPR compared to sham rats from d1 to d3 as shown by the longer time spent to remove the tape (Figure 2(b)). At later timepoints CA/CPR rats showed a trend towards persistence of deficit even if not significant when compared to sham.

Analysis of spontaneous locomotor activity, evidenced physiological cyclical variation every 12 hours, scoring lower during the light phases (supplemental material) and higher during the dark phases, as expected. Groups showed no difference in spontaneous locomotor activity at baseline (Figure 2(c)) as well as during the light phase (supplemental material). During the dark light phase CA/CPR rats showed significant reduction in locomotor activity from d1 to d9 (Figure 2(c)) and recovered at later times with no difference compared to sham.

At d13, the open field test revealed a significant impairment in exploratory behavior of CA/CPR rats measured as lower number of rearings compared to sham (Figure 2(d)) with no change in inner (inversely related to anxious behavior) and outer (related to motor activity) crossed squares as well as time spent in internal, external squares and in first corner (details in the supplemental material).

## MRI reveals brain edema and white matter (WM) damage after CA/CPR

**Figure 3.**
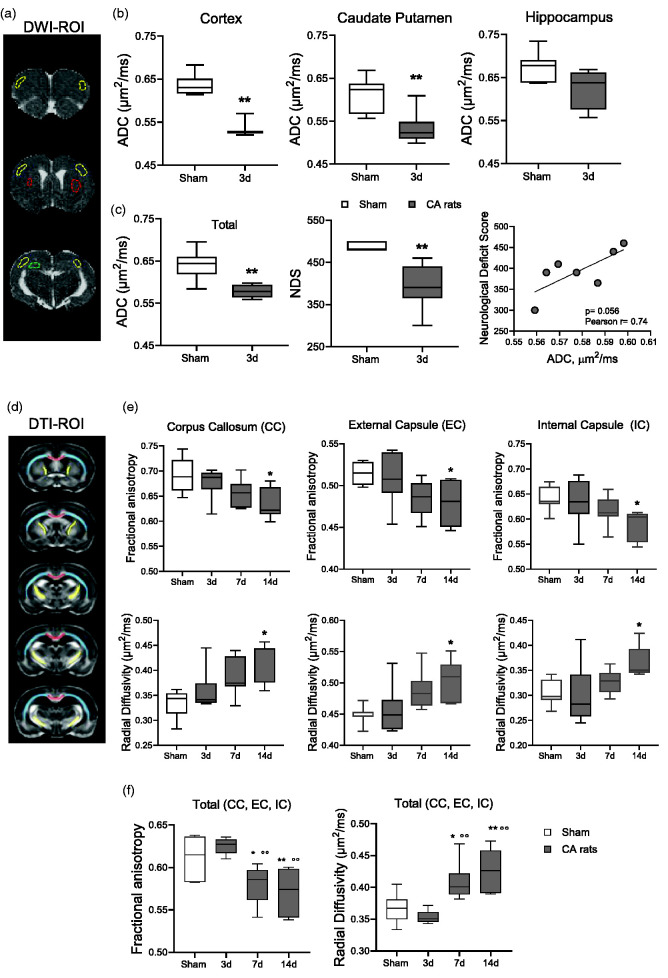
MRI analysis. (a) Representative brain coronal sections by apparent diffusion coefficient (ADC) maps. Hypo-intense regions depict restricted water diffusion (cytotoxic edema) in cortex (yellow), caudate putamen (red) and hippocampus (green) of a CA rat after CA/CPR. (b) Quantitative analysis of ADC maps of CA and sham rats. (c) Quantification of ADC maps in edematous areas (mean of cortex, caudate putamen, hippocampus), neurological deficit score and correlation between ADC maps and neurological deficit score in CA rats (Pearson r = 0.74, p = 0.056). Mann-Whitney. *p < 0.05, **p < 0.01 vs sham. n = 8–7. (d) Drawings represent the rostrocaudal ROIs selection for diffusion tensor imaging (DTI-MRI) sequences in corpus callosum (CC, red), external capsule (EC, light blue) and internal capsule (IC, yellow) of the rat brain. (e) Quantitative analysis of DTI-MRI parameters in CA and sham rats and (f) Fractional anisotropy and radial diffusivity in WM tracts (mean of CC, EC, IC). One-way ANOVA for repeated measurements followed by Tukey's post hoc test. *p < 0.05, p < 0.01 vs sham. °°p < 0.01 vs 3d. n = 8-7-7-8.

Three days after CA/CPR, ADC maps showed hypointense regions in cortex (Cx), Caudate putamen (CPu) and to a lesser extent in the hippocampus. (Figure 3(a)). Compared to sham, ADC maps values were significantly lower in Cx and Cpu of CA/CPR rats, while no significant changes were detected in the hippocampus (Figure 3(b)). At d3 after CPR, a strong positive correlation was observed between the ADC values in edematous brain areas (Cx, CPu and hippocampus) and neurological function assessed as NDS (Figure 3(c)).

DTI-MRI analysis was performed in WM regions (CC, EC and IC Figure 3(d)). Compared to sham, CA/CPR significantly reduced fractional anisotropy (FA) in the CC, EC, IC at d14 (Figure 3(e)). Radial diffusivity (RD) significantly increased in WM regions at d14 in CA rats (Figure 3(e)). Analysis of the axial diffusivity (AD) indicated a significant increase in EC and IC at d14 after CA (details in the supplemental material). Evidence of degenerative processes in WM after CA were further confirmed and extended when FA and RD in whole WM tracts (mean of CC, EC, IC). CA/CPR significantly decreased FA and increased RD at d7 and d14 (Figure 3(f)).

## Histology reveals progressive neuropathology after CA/CPR

**Figure 4.**
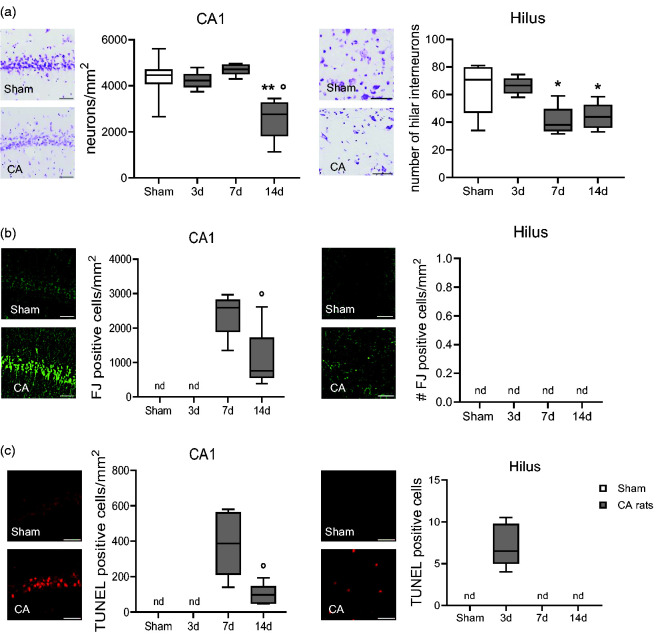
Representative images and quantification of cresyl violet stained neurons (a), Fluoro-Jade (FJ, b) and TUNEL positive cells (c) of CA and sham rats. One-way ANOVA followed by Tukey's post hoc test (a). *p < 0.05, **p < 0.01 vs sham: °p < 0.05 vs 7d. Unpaired t-test (b, c). °p < 0.05, °°p < 0.01 vs 7d. n = 6-5-6-8. (n.d., not detectable). Scale bar, 50 μm.

Compared to sham, neuropathological studies at d3, d7 and d14 after CA/CPR evidenced reduced neuronal density in CA1 pyramidal cell layer at d14 and in hilar interneurons at d7 and d14 (Figure 4(a)), while there was no detectable neuronal loss in the Cx and CPu (details in the supplemental material).

Quantitative analysis of Fluoro-Jade (FJ)^13^ stained neurons revealed degenerating neurons in the CA1 pyramidal cell layer of the hippocampus at d7 and d14 after CA. FJ reactivity was undetectable in sham rats in CA1 and hilus at d3 (Figure 4(b)), and in Cx and Cpu at all time points (data not shown).

At d3 after CA/CPR, apoptotic TUNEL-positive cells were present in the hilus, and undetectable at later time points (Figure 4(c)). Apoptotic cells were present in CA1 region of hippocampus at d7 and d14, indicating the persistence of dying cells. TUNEL staining was undetectable in any sham rats (Figure 4(c)), at all-time points as well as in the CA3 region of hippocampus (data not shown).

**Figure 5.**
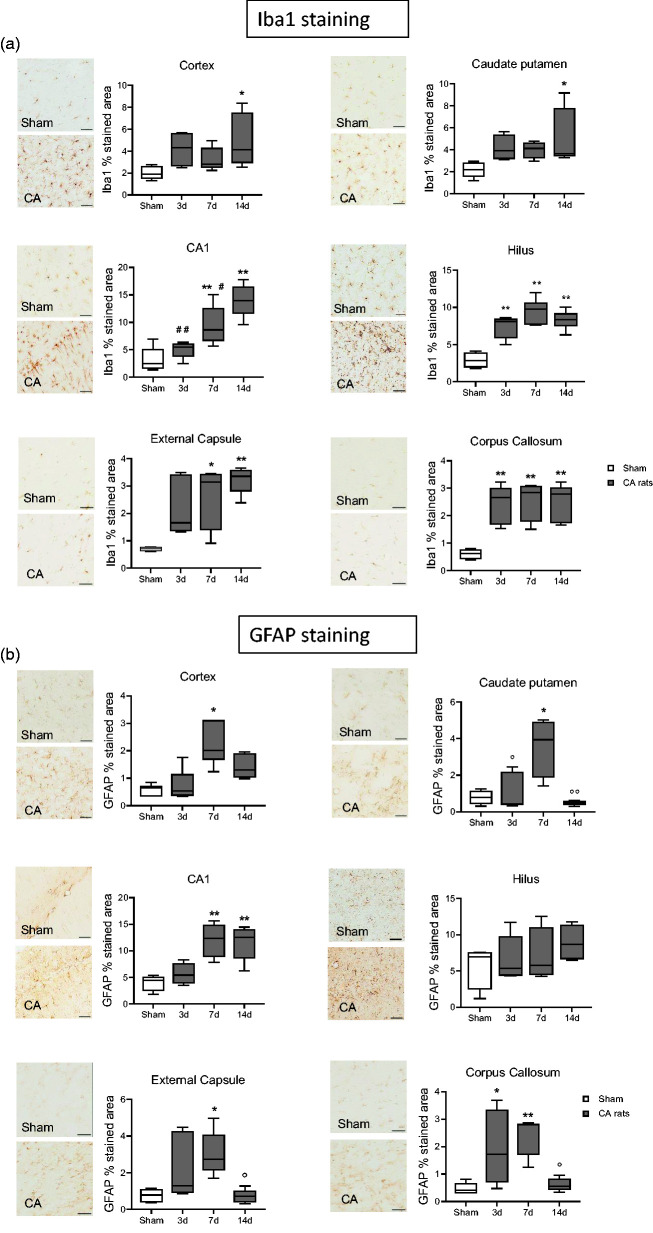
Representative micrographs of Iba1 (a) and GFAP (b) and their quantifications in cortex, Cpu, CA1 hippocampal region, hilus, external capsule and corpus callosum of CA and sham rats. One-way ANOVA followed by Tukey's post hoc test. *p < 0.05, **p < 0.01 vs sham; ^#^p < 0.05, ^##^p < 0.01 vs 14d; °p < 0.05, °°p < 0.01 vs 7d. n = 6-5-6-8. Scale bar = 50 µm.

Immunohistochemical studies found that CA/CPR is associated with microgliosis and astrogliosis, marked by increased area of staining for Iba1 and Glial fibrillary acidic protein (GFAP). Iba1, was expressed at every timepoint considered, and in sham rats (Figure 5(a)). Iba1 immunoreactivity significantly increased in Cx and CPu at d14 and increased in the CA1, hilus, EC, and CC at all timepoints in CA/CPR rats (Figure 5(a)). These changes were associated with astrogliosis marked by significant increase in GFAP area staining in Cx at d7, in Cpu at d3 and d7, in CA1 and CC at d7 and d14, and in EC at d7 (Figure 5(b)).

**Figure 6.**
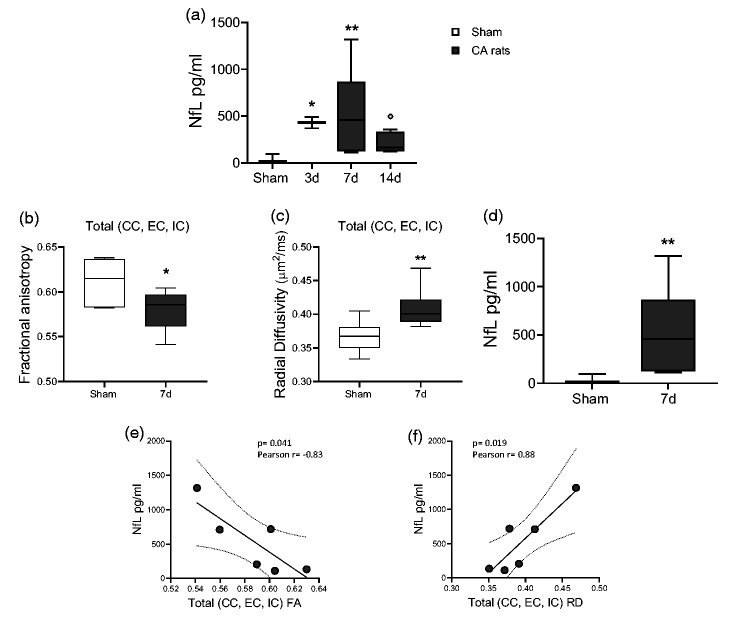
(a) Plasma levels of neurofilament light (NfL) after CA/CPR. One-way ANOVA followed by Tukey's post hoc test. *p < 0.05, **p < 0.01 vs sham; °p < 0.05, vs 7d. n = 6-5-6-6. (b, c) Quantitative analysis of diffusion tensor imaging (DTI-MRI) parameters in whole white matter tracts (mean CC, EC, IC). (d) Levels of neurofilament light (NfL) at 7d after CA/CPR. (e, f) Correlation between total FA (e), total RD (f) and levels of NfL at 7d after CA/CPR (in (e) Pearson r = −0.83, p = 0.041. In (f) Pearson r = 0.88, p = 0.019). One-way ANOVA followed by Mann Whitney (b, c) and Student t-test (d). *p < 0.05, **p < 0.01 vs sham. n = 6.

Axonal and neuronal damages were also assessed by measuring the plasma NfL leakage. Plasma levels of NfL were significantly higher at d3 and d7 after CA/CPR as compared to sham and remained elevated up to d14 (Figure 6(a)).

When FA in whole WM tracts (CC, EC, IC, Figure 6(b)) was correlated with NfL levels of CA rats at d7 (Figure 6(d)), an opposite correlation was observed between FA and the NfL levels (Figure 6(e)). Similarly, a positive correlation was observed between RD in WM tracts (Figure 6(c)) and NfL levels at d7 (Figure 6(f)). No correlation was found between whole WM tracts and NfL levels at d3 and d14.

## Discussion

Overall, our study describes in detail, for the first time, the progression of CA-induced neuropathology, MRI abnormalities, blood biomarker elevations, and neurological deficit up to 14d after resuscitation and introduces new clinically translatable outcome parameters that could be used to test therapeutic interventions in the rat.

We used a well-established model of CA and CPR in the rat that has been used to investigate new treatments to improve outcome and to investigate mechanisms of organ injury and brain dysfunction after CA.^8^ This study shows that CA triggers a progressive neuropathological injury that spreads across the brain with distinctive spatial and temporal features. *In vivo* longitudinal MRI studies evidenced the appearance of brain pathology with involvement of WM structures and ongoing neuroinflammation as proved by histology up to 14d after resuscitation. In addition, a late neurodegeneration, apoptosis, neuronal loss in selected brain areas was evidenced and reflected by increased NfL plasma levels and persistent behavioral and functional dysfunction.

NDS and the TRT tests have been used in several studies yielding mixed results.^10,14–16^ Available data reported early sensorimotor deficits with a recovery of function by approximately 1 week post CA, or showed a weak sensitivity to detect neurological dysfunction; thus their ability to disclose a treatment effect at sub-acute stages still needs to be verified.^14,16^ In this study, after 8 minutes of untreated CA, NDS and the TRT clearly distinguished CA rats from sham ones, although only transiently. Indeed, differences were significant only up to 3d after CA while at 7 and 14d only a small deficit persisted, in contrast with concurrent MRI and histopathology analyses and NfL levels. At 14d after CA, instead, a reduction in rearing behavior was evident in CA rats, thus suggesting that evaluation of functional impairment in the exploratory behavior by the open field test could be preferred over NDS and TRT for evaluation of long-term outcome. As shown in models of brain ischemia, exploratory behavior can provide information on modifications induced by a selected treatment.^17^

A drawback of the open field test is the existing little assessment on gait and functional use of the animal’s limbs, which could be affected after global cerebral ischemia.^18^ When analyzing longitudinal data obtained on automated cages during the dark phase, a significant reduction in locomotor activity was evident from day 2 to day 9 in post-CA rats compared to sham. This effect was not evident during the light phase. Thus, it seems unlikely that sensorimotor or exploratory performance was affected by reduced locomotor activity in any of the tests utilized during the light phase. Since locomotor activity was monitored in single housing, this could have exacerbated anxiety and performance on the open field test. However, monitoring of spontaneous activity in rodents^19^ is not feasible in paired housing. Our observations confirm that no gold standard exists for behavioral test selection in rodents after CA. Tests should complement each other for assessment of global neurological status, sensorimotor coordination, locomotor activity and cognitive impairment.^18^

Hypoxic ischemic brain injury can be followed by cerebral edema in the post-CA period.^20^ Restricted water diffusion outside the cells reflecting microenvironmental changes such as cytotoxic edema, can be quantified by MRI-ADC value of each voxel thus limiting subjectivity. While clinically the presence of cerebral edema for prognostication after CA has rapidly gained interest,^20^ in animal models the extent of edema is helpful to monitor the progression of an intervention.^19,21^ Hence, we used MRI ADC maps to investigate brain edema in hypo-intense brain areas. At 3d after CA a reduction of water diffusion was observed in the Cx, CPu and to a small extent in the hippocampus of CA rats. Despite its known sensitivity to post-CA injury, the hippocampus failed to show consistent ADC changes. This finding may result from the presence of concurrent vasogenic edema, which could have masked diffusion restriction by elevating the ADC value, a phenomenon termed “pseudonormalization”.^22^ When neurological score was correlated with ADC values in the edematous brain areas, a positive correlation was found, i.e. for reduced ADC value and worst neurological score. These observations are in line with clinical findings showing that at acute stages, MRI ADC maps features could be associated with outcomes in patients resuscitated from CA.^20^

Histopathological analysis at d3 post-CA showed apoptotic cells and loss of interneurons at 7 and 14d in the hilus. This effect was accompanied by neurodegeneration at 7 and 14d, apoptosis in at 7 and 14d and neuronal loss at 14d in CA1.

Hilar inhibitory interneurons, are particularly susceptible to cell death after brain injury and this could explain the early damage in this brain area. Loss of inhibitory GABAergic interneurons may alter excitation-inhibition balance in the hippocampus and may promote epileptiform activity after brain injury, condition that we occasionally observed in some animal.^23–25^

As previously shown^26–28^ we also observed delayed degenerating and dying cells in hippocampal subfields. Notably, even if this deserves more attention in future studies, the impaired exploratory behavior observed at 14d after CA, was coherent with the timing of neuronal cell death in CA1 subfield of hippocampus. A few studies addressing neuronal damage in CA rats report earlier neuronal loss and involvement of CPu and Cx. These studies differ for the CA model employed, the time point explored (i.e. 5d after CA that was not investigated in this study), and/or rats strain and age.^26–29^ Of note, in this study, TUNEL as evidence of apoptosis and F-J staining for evidencing neurodegeneration appear to anticipate, at d3 and d7 respectively, the neuronal loss evidenced subsequently, i.e. d7 and d14, by cresyl violet staining in the subfields of hippocampus.

Microglia are the major cellular contributors to post-ischemic inflammation. They rapidly respond to injury or alterations of microenvironment with morphological changes and production of factors that contribute to neurological outcome by promoting injury and/or lesion repair.^12^ Glial fibrillary acidic protein (GFAP) is the most widely used marker of reactive astrocytes; increased GFAP is a response to injury and, a sensitive indicator detectable even in the absence of overt neuronal death.^30^ Furthermore, suppression of astrogliosis can promote brain functional recovery in ischemic stroke.^31^

We found neuroinflammatory processes with active microgliosis and astrogliosis in the CA1 and Hilus subfields of hippocampus where neurodegeneration and apoptosis were present, in edematous areas with low MRI ADC values as Cx and Cpu, and in WM tracts (EC and CC). These observations confirm our behavioral and MRI ADC data suggesting the emergence and persistence of brain pathology with microenvironmental changes in the acute and sub-acute phase after CA. Although the neuroinflammatory phenotypes were not investigate in our study, our observations reinforce the notion that brain damage after CA, can be enhanced by this additional mechanism.^27,32^

MRI-DTI allows to assess the integrity and organization of brain WM tracts and axonal and myelin damage in humans.^33^ Here, we documented WM damage after CA with FA values significantly lower than in controls in the CC, EC and IC together with increased RD at 14d after CA, indicating loss of microstructural organization, quantified by the loss of directionality in the diffusion of water molecules, suggesting alteration of the axonal and myelinic component of fiber structure. WM damage was further confirmed and extended by altered FA and RD in whole WM tracts (mean of CC, EC, IC) found at d7 and d14.

Whether these changes are the consequence of Wallerian degeneration or due to secondary evolving pathologies detrimental to axons still needs to be clarified. Our results close resembled the clinical findings described in patients resuscitated from CA,^33^ further confirming the additional value of our model.

We also observed a sustained neuroinflammation with activated microglia and astrocytosis in the WM tracts CC and EC. Myelin loss and concurrent activation of microglia and astrocytes has been supported histologically after global cerebral ischemia as well as the critical role of microglia activation in WM injury via complement C3-C3aR pathway after brain hypoperfusion.^32,34^ This evidence substantiates our observations and may underlie the neurobehavioral impairment detected at d14 after CA.

NfL is an intermediate filament protein that is a component of the cytoskeleton of neurons and is abundantly expressed in axons. Increased blood concentrations of NfL has more recently appeared as the most consistent predictor of neurological outcome in post-CA patients^35^ and in preclinical model of brain injury.^36^ In our study, NfL plasma levels increased at 3, 7 and 14d after CA. Comparing the timing of NfL to histopathology and MRI abnormalities, we speculate that at d3, NfL plasma levels could be related to MRI-ADC edema, apoptosis and neuroinflammation observed in hippocampus, Cx, CPu, and WM tracts. NfL increase appears to peak at d7 after CA when neurodegeneration, apoptosis, neuroinflammation, neuronal loss, and the whole WM tracts damage were clearly evident. Seven days later, at d14, NfL levels decreased, parallel with a concurrent reduced neurodegeneration, apoptosis, astrogliosis, but nearly stable neuronal loss and WM tracts damage. In this frame, NfL plasma levels at d7 significantly correlated with FA as well as with RD values in CA rats. These results adds relevance to the translational value of our observations and to the potential impact of our model in the clinical settings.^37^ Importantly, NfL appears as a valid circulating biomarker whose pattern reflects the progression of imaging, and histopathological damage. Thus, NfL assay might be a useful indicator of the severity of neurological damage as well as readout of potential protective interventions.

Several limitations need to be acknowledged in the interpretation of the findings. First, only male rats were used. Indeed, estrogens have been reported to affect the ischemic outcome in experimental models.^38^ Nevertheless, the hormonal contribution to the ischemic brain damage was beyond the aim of this work. Second, our model employed a duration of untreated VF of 8 min which probably resulted in a mild/moderate brain injury as reflected by the transient NDS and brain edema observed. Nevertheless, this duration of untreated VF represents a good compromise between probability of ROSC and the possibility to generate a standardized and now well characterized brain injury that is mild but valid to test the therapeutic effects of new treatments.^8,39^ Future studies with more prolonged CA durations are needed. Third, we did not discriminate the role of primary injury due to CA/CPR from those of secondary injury due to reperfusion and concomitant events i.e. seizures, microcirculatory deregulation, systemic inflammation, etc., which might have accounted especially for the late neurodegeneration and apoptosis observed in selected brain areas. Fourth, thiopental is known to play neuroprotective effects and thus might have mitigated the final CA/CPR-related brain injury. Nevertheless, thiopental has been already employed in rat models of CA/CPR without interfering with the development of post-CA neurological injury and/or with the effect of the experimental treatments. In addition, the dosage of thiopental used in this study was the same among the CA/CPR groups (supplemental material). Additionally, it is worth noting that behavioral tests, such as fear conditioning or the Morris water maze (MWM), were not included in our study but should be considered in future research to assess hippocampal-dependent learning and memory. Finally, no treatments to mitigate post-CA brain injury were tested in this study.

## Conclusion

Our study shares light on the consequences of CA on neurological functions, brain micro environmental and microstructural damage underlying sensorimotor impairments up to 14d after CA. The combination of functional, histological, blood biomarker and imaging data presented here provide unique insights into the complex brain response that follows CA and present a valid model to investigate neurological injury and dysfunction in translational studies employing the rat model of CA.

## Supplemental Material

sj-pdf-1-jcb-10.1177_0271678X241255599 - Supplemental material for Evolution of brain injury and neurological dysfunction after cardiac arrest in the rat – A multimodal and comprehensive model Supplemental material, sj-pdf-1-jcb-10.1177_0271678X241255599 for Evolution of brain injury and neurological dysfunction after cardiac arrest in the rat – A multimodal and comprehensive model by Carlo Perego, Francesca Fumagalli, Francesca Motta, Marianna Cerrato, Edoardo Micotti, Davide Olivari, Daria De Giorgio, Giulia Merigo, Angelo Di Clemente, Alessandra Mandelli, Gianluigi Forloni, Luigi Cervo, Roberto Furlan, Roberto Latini, Robert W Neumar and Giuseppe Ristagno in Journal of Cerebral Blood Flow & Metabolism

## Data Availability

The datasets generated and analyzed during the current study can be found at: https://zenodo.org/communities/irfmn-irccs.
