# Supplementary material for: Evolution of brain injury and neurological dysfunction after cardiac arrest in the rat – A multimodal and comprehensive model
Source: J Cereb Blood Flow Metab. 2024 May 21:0271678X241255599. Online ahead of print. doi: 10.1177/0271678X241255599 (PMC11542119; doi:10.1177/0271678X241255599)
Supplement: sj-pdf-1-jcb-10.1177_0271678X241255599 - Supplemental material for Evolution of brain injury and neurological dysfunction after cardiac arrest in the rat – A multimodal and comprehensive model [file sj-pdf-1-jcb-10.1177_0271678X241255599.pdf]

SUPPLEMENTAL MATERIAL

Evolution of brain injury and neurological dysfunction after cardiac arrest in the rat – a multimodal and comprehensive model

Carlo Perego BS[1], Francesca Fumagalli PhD[1], Francesca Motta MSc[1], Marianna Cerrato MSc[1], Edoardo Micotti PhD[2], Davide Olivari PhD[1], Daria De Giorgio MSc[1], Giulia Merigo[3] MSc, Angelo Di Clemente BS[2], Alessandra Mandelli BS[4], Gianluigi Forloni PhD[2], Luigi Cervo PhD[2], Roberto Furlan MD, PhD[4], Roberto Latini MD[1], Robert W. Neumar,[5] Giuseppe Ristagno MD, PhD[3,6].

[1] Department of Acute Brain and Cardiovascular Injury, Istituto di Ricerche Farmacologiche Mario Negri IRCCS, Milan Italy.

[2] Department of Neuroscience Istituto di Ricerche Farmacologiche Mario Negri IRCCS Milan Italy.

[3] Department of Anesthesiology, Intensive Care and Emergency, Fondazione IRCCS Ca' Granda Ospedale Maggiore Policlinico, Milan, Italy

[4] Clinical Neuroimmunology Unit Division of Neuroscience Institute of Experimental Neurology - INSpe San Raffaele Scientific Institute Milan Italy.

[5] Department of Emergency Medicine and Max Harry Weil Institute for Critical Care Research and Innovation, University of Michigan, Ann Arbor, Michigan, USA

[6] Department of Pathophysiology and Transplantation, University of Milan, Milan, Italy

Correspondence to:

Francesca Fumagalli
Department of Acute Brain and Cardiovascular Injury
Mario Negri Institute for Pharmacological Research IRCCS
Via Mario Negri 2, 20156 Milano, Italy
Phone: +39 02 3901 4622
Fax: 02-33200049
Email: francesca.fumagalli@marionegri.it

Materials and methods

Animals

We used male Sprague-Dawley ex-breeder rats (weighting 479±15 g, Envigo RMS srl, Italy). Animals were housed in certified specific pathogen-free vivaria with constant temperature (21±1°C) and relative humidity (60±5%), a 12 h light–dark cycle and free access to pellet food and water. VRF1 (SDS) Rodent Diet.

Procedures involving animals were conducted at the Istituto di Ricerche Farmacologiche Mario Negri IRCCS, which adheres to the principles set out in the following laws, regulations, and policies governing the care and use of laboratory animals: Italian Governing Law (D.lgs 26/2014; Authorization n.19/2008-A issued 6 March 2008 by Ministry of Health); Mario Negri Institutional Regulations and Policies providing internal authorization for persons conducting animal experiments (Quality Management System Certificate-UNI EN ISO 9001:2008-Reg. No. 8576-A); the NIH Guide for the Care and Use of Laboratory Animals (2011 edition) and EU directives and guidelines (EEC Council Directive 2010/63/UE). They were approved by the Mario Negri Institute Animal Care and Use Committee that includes ad hoc members for ethical issues, and by the Italian Ministry of Health (Decreto n.895/2020 PR issued September 28, 2020). All procedures regarding the study design, animal experiments, statistical analysis, and data reporting fulfil the criteria of the ARRIVE (Animal Research: Reporting of In Vivo Experiments) guidelines (https://www.nc3rs.org.uk/arrive-guidelines) with a commitment to refinement, reduction and replacement, avoiding any unethical and unjustified use of animals throughout the entire experiment. We used only male rats since estrogens affect the ischemic outcome in experimental models (Carswell HVO, Macrae IM, Farr TD. Complexities of oestrogen in stroke. *Clin. Sci. Lond. Engl. 1979*. 2009;118:375–389). The hormonal contribution to the ischemic brain damage was beyond this work's aim, which was to explore the ischemic injury that follows CA and CPR.

Animal preparation

Before surgery, animals were fasted overnight, with free access to water. They were anesthetized by intraperitoneal (IP) injection of thiopental l (50 mg/kg). Additional doses of thiopental (10 mg/kg) were given at intervals of approximately 40 minutes or when required to maintain anaesthesia. Total doses (mg/kg) of administered thiopental in CA/CPR groups were (mean±SD): 66,9±8,5 for d3; 71,0±7,4 for d7; 68,2±6,4 for d14, with no statistical difference among groups. Ampicillin (50 mg/kg) was injected intramuscularly (IM) as prophylaxis after induction of anaesthesia. Animals were then instrumented for hemodynamic measurements and induction of cardiac arrest (CA) according to our established model of electrically induced CA and CPR. Briefly, the trachea was orally intubated with a 14-gauge cannula. A PE-50 catheter was advanced into the descending aorta from the left femoral artery for measurements of arterial pressure (systolic, median and diastolic arterial pressure: SAP, MAP and DAP) and blood sampling. Through the left external jugular vein, another PE-50 catheter was advanced into the right atrium for measurement of right atrial pressure (RAP) and for the administration of epinephrine. Aortic and right atrial pressures were measured with reference to the mid-chest with conventional external pressure transducers. A 3-Fr PE catheter was advanced through the right external jugular vein into the right atrium. A pre-curved guide wire supplied with the catheter was then advanced through the catheter into the right ventricle for inducing CA. All catheters were flushed intermittently with saline containing 2.5 IU/mL of bovine heparin. A conventional lead II electrocardiogram (ECG, DataQ, Akron, OH) was continuously monitored. Temperature was monitored with the aid of a rectal probe (Physitemp instrument INC. Clifton, NJ) and maintained at 37±0,5 ºC. The same anaesthetic and surgical procedures were performed for the sham-operated rats excluding cardiac arrest and cardiopulmonary resuscitation.

Cardiac arrest (CA) and cardiopulmonary resuscitation (CPR) procedures

Rats (n=43) were randomized[1] to CA/CPR or sham surgery. Ventricular fibrillation (VF) was electrically induced with progressive increases in 60-Hz current to a maximum of 4 mA delivered to

the right ventricular endocardium. The current flow was maintained for 3 min to prevent spontaneous defibrillation. Animals were subjected 8 minutes of untreated VF following by precordial compression (PC) with a pneumatically driven mechanical chest compressor. The PC depth was adjusted to ensure *coronary perfusion pressure* (CPP) at least of 20-25 mmHg. The PC rate was 200/min with equal compression-decompression. From the start of PC, animals were mechanically ventilated at a frequency of 50/min with tidal volume 0.6 mL/100g and FiO_2 1.0. A single dose of epinephrine (0.02 mg/kg) was injected into the right atrium 2 min after the start of PC. After 8 minutes of CPR, resuscitation was attempted with up to three 2-joule (J) defibrillations (CodeMaster XL, Philips Heartstream). ROSC was defined as the return of supraventricular rhythm with MAP > 50 mmHg for at least 5 minutes. If ROSC did not occur, two more 1minute cycles CPR were done with counter-shock. After ROSC, mechanical ventilation was maintained at FiO_2 1 for 1h post-resuscitation, then continued with FiO_2 0.21. Blood samples were serially collected from the femoral artery cannula 15 minutes before CA (BL), and 1 and 3 hours after ROSC. Ampicillin (50 mg/kg) was injected intramuscularly as prophylaxis after induction of anaesthesia. Three hours after ROSC, animals were observed for additional 2 hours in the operating room prior to be returned to their cages. Ampicillin (50 mg/kg intramuscular) and buprenorphine (0.16 mg/kg) were given to prevent infection and pain. CA/CPR was performed on 32 rats. Four CA/CPR rats were excluded because of spontaneous ROSC during the 8 minutes of untreated VF. Post-ROSC mortality at 72h was 38%. Sham-operated rats (n=8) received the identical anesthesia and surgical procedure without CA induction. Since the rapid decrease in body temperature in the rat during the cardiac arrest phase might result in neuroprotective effects, aiming to maximize brain injury after CA/CPR, we maintained the body temperature not lower than 35.5 °C during CA/CPR.

Measurements

Recorded hemodynamics. Briefly, ECG, aortic pressure, and right atrial pressure were continuously monitored for up to 3 hours after ROSC on a personal computer–based data acquisition system

supported by CODAS hardware and software (DataQ, Akron, OH). Coronary perfusion pressure was calculated in the same time range as the difference between time- coincident diastolic aortic and right atrial pressures.

Neurological Assessment

Neurological deficit scores (NDS)[2] and tape removal test (TRT)[3] were used to assess sensorimotor deficits at the times indicated in figure 1. Here (Figure S3) we report results obtained with an abbreviated version of the NDS test by Neumar et al., 1995 [2], and shown in Figure 2A, main text. Briefly, NDS rated level of consciousness, respiration, motor and sensory functions, and overall behaviour (score 0 for sham, to 500, worst performance) [2]. NDS values assessed at d7 in one CA rat were exclude because of ADC and DTI image artefact. TRT measured the time until the animal removed adhesive tapes (adhesive tape size was 10-mm by 12-mm) from both their fore paws. The test was truncated at 180 sec[3].

Locomotor Activity

Rats spontaneous motor activity was evaluated during light and dark phase of day for 3 days before and 12 days after the cardiac arrest or sham surgery (light phase between 7 AM and 7 PM; dark phase between 7 PM and 7 AM). Rats were kept in individual transparent cages (42 x 28 x 21 cm, length x width x height) with sawdust bedding. Each cage was placed between metal frames (54 x 50 x 37 cm) holding two sets of parallel photo beams, crossing the cage 3 cm above the floor (Multiple Activity Cage, Ugo Basile, Comerio, Varese, Italy). The device counts the number of movements by recording the number of infrared beam interruptions. Rats were randomly allocated to the transparent cage and metal frames and baseline motor activity counts were first automatically recorded using dedicated software (Ugo Basile) as the number of beam breaks in a 30 min time bin over a period of 12 (light) + 12 (dark) hours.

Open-field activity

To see whether sham and CA rats exhibited changes in exploratory and motor activity, animals were tested in an open field 13d after the CA or sham surgery. The apparatus was a square box (100×100 cm) with 40-cm-high plastic walls. The floor consisted of a sheet of white plastic painted with a black grid dividing the field into 25 (5×5 cm) equal squares. Rats were tested under dim illumination provided by a 60 W lamp placed 1 m above the apparatus and pointed toward the ceiling. Rats were individually placed into the center of the open field and their behavior was-video-recorded during 5 minutes session. Animals were scored for the time (s) spent to reach the corner of the open field, the number of internal and external square crossing (rat enter and the number of rearing were later scored by two observers unaware of the treatments. An animal was considered to enter a square with all four paws. We used Ethovision XT 5.0 (Noldus Information Technology, Wageningen, NL) recording software to record the time. After each use the apparatus was completely cleaned with 70% ethanol and carefully dried.

Brain Magnetic Resonance Imaging acquisitions and analysis

Brain imaging was done on a 7T small-bore animal scanner (BioSpec®; Bruker, Ettlingen, Germany) running ParaVision 6.01 (Bruker, Ettlingen, Germany). To characterize post-CA brain injury, diffusion-weighted imaging (DWI) and diffusion tensor imaging (DTI) sequences were acquired for quantification of cerebral edema and white matter damage respectively at 3, 7 and 14 days after CA. Anaesthetised rats (isoflurane 1.5-2 vol% in an N_2O/O_2 (70%/30%) were positioned in the magnet at the timepoints indicated in Fig. 1. Respiratory frequency was monitored throughout the experiment and body temperature was maintained at 37°C with a heating pad.

The severity of white matter damage was quantified by comparing the values of fractional anisotropy (FA) and diffusivity (radial (RA), axial (AD) obtained from DTI sequences. The apparent diffusion coefficient (ADC) was used as a quantitative measurement of water diffusion changes in the brain.

Intracerebral cytotoxic edema reduces water diffusivity and this corresponded to a reduction in ADC values.

Diffusion weighted echo-planar images (TR/TE=7000/31.2 ms, slice thickness= 0.6 mm, in-plane resolution = 0.156x0.156 mm^2) covering the brain from olfactory bulbs to the beginning of cerebellum, were adopted to obtain the apparent diffusion coefficient (ADC)-maps. Diffusion-encoding was applied in 3 orthogonal directions with b values of 700 s/mm^2, respectively. ADC-maps were calculated on a pixel-by-pixel basis using the model function: $\ln(S(b)/S0)=-b\cdot ADC$, where S(b) is the measured signal intensity at a specific b value (b) and S0 the signal intensity in the absence of a diffusion gradient (b = 0). The different ADC values per region were calculated. ADC maps in sham operated rats revealed a mean grey matter value of 0.62±0.04. In line with clinical studies, cytotoxic edema was revealed in images from CA rats when threshold was performed with a lower cut-off value of 0.52 $\mu m^2/msec$[4,5]. An expert operator blind to experimental condition extracted the ADC values in thresholded map using freely available ITK-SNAP software. Brain areas from sham and CA rats were selected manually by a trained expert following the rat brain atlas (Paxinos G, Watson C. The rat brain in stereotaxic coordinates. New York, NY: Academic Press; 2005). The average ADC value within each region was computed for each animal. Group average ADC values were reported for each region and the mean of the analysed regions was reported as total.

Diffusion tensor imaging (DTI): echo-planar imaging sequences were acquired (TR/TE=6000/21.5 ms, slice thickness= 0.6 mm, in-plane resolution = 0.178x0.178 mm^2) with the same geometry as DWI images. Diffusion encoding b factors of 700 s/mm^2 were applied along 19 isotropic directions and two B0 unweighted images for each repetition. The diffusion tensor was computed using FSL software. A group mean full tensor template was first created using a population-based DTI atlas construction algorithm that adopts a tensor-based registration procedure embedded in the DTI-TK software library. The average template was then resampled to an in-plane resolution of 100 × 100 μm^2 and slice thickness 0.2 mm, and skeletonized. FA images of all subjects were normalized to the mean template with a diffeomorphic transformation and the transformations were applied to all the

DTI diffusivity metrics (radial, axial), which were warped to the mean skeleton for region of interest (ROI)-based analysis.

Qualitative MRI variables used from DTI sequences were axial (AD), radial diffusivity (RD) and fractional anisotropy (FA). Axial diffusivity represents the rate of water diffusion along the principal axis parallel to the main vector of the white matter fibers, while radial diffusivity indicates the rate of diffusion perpendicular to the main vector. These values reflect respectively, axonal degeneration and myelin loss. Fractional anisotropy provides instead a cumulative direction of water diffusion, suggesting that lower value of FA indicates a loss of directionality of water molecule diffusion in the white matter tracts representing microstructural damage of the white matter fibers. ROIs were the corpus callosum (CC), the external capsule (EC), the internal capsule (IC) and were selected manually on the reference template by a trained expert following the rat brain atlas (Paxinos G, Watson C. The rat brain in stereotaxic coordinates. New York, NY: Academic Press; 2005).

ADC values assessed in one CA rat at d3 were excluded due to image artefact as well as NDS value of the same animal (Figure3). DTI values assessed in one CA rat at d7 were excluded because of poor quality DTI images (figure 3).

Tissue processing for histopathological analysis

At 3,7 and 14 d after sham or CA surgery, rats were euthanized with an intraperitoneal injection of pentobarbital sodium (150 mg/kg). Blood samples were collected from the descending aorta artery in 3K- EDTA tubes. Rats were then perfused via the ascending aorta with cold phosphate-buffered saline (PBS), 0.1 mol/l, pH7.4, followed by chilled paraformaldehyde (4%) in PBS. After decapitation the brains were carefully removed from the skull and post-fixed for 6 h at 4 °C, and then transferred to 30% sucrose in 0.1 mol/l PBS for 24 h until equilibration. The brains were frozen by immersion in isopentane at −45 °C for 3 minutes before being sealed into vials and stored at −80 °C until use.

Coronal brain cryosections 20 μm thick were cut serially at -20°C (CM1850UV Leica Biosystems, Germany) and stored at 4 °C in a solution of glycerol:PBS (1:1). Brain slices from sham and CA rats were matched for anteroposterior level. Neuronal cell loss was evaluated on cresyl violet stained sections.

Immunohistochemistry

Immunohistochemistry was done on 20 μm brain coronal sections incubated overnight at 4°C with anti-Iba1 (1:200; Wako, Neuss, Germany) to detect microglia macrophage activation and with primary monoclonal antibody mouse anti-mouse glial fibrillary acid protein (GFAP, 1:2000, Millipore, Billerica, MA, USA). Biotinylated secondary antibodies (1:200, Vector Laboratories, CA, USA) were used. Positive cells were stained by reaction with 3,3 diaminobenzidine tetrahydrochloride (DAB, Vector laboratories, CA, USA). Negative control studies, without the primary antibody, were performed in parallel.

Slice selection and image acquisition

Three brain coronal sections per rat at +4.68, +0.60 and -3.48 mm from bregma (Paxinos G, Watson C. The rat brain in stereotaxic coordinates. New York, NY: Academic Press; 2005), were used to quantify neuronal cells and immunostainings. Slices from sham and CA rats were matched for anteroposterior level. The entire brain sections were acquired at 20X with a pixel size of 0.346 μm (10× magnification, with a pixel size of 0.694 μm for F-J and TUNEL) by an Olympus BX-61 Virtual Stage microscope equipped with motorized platform (Olympus, Hamburg, Germany) and digitized. Acquisition was done over 10 μm thick stacks, with a step size of 2 μm. The different focal planes were merged into a single stack by mean intensity projection to ensure consistent focus throughout the sample.

Definition of regions of interest (ROI) and image quantification

Images were analyzed using Fiji software (https://fiji.sc/). Neuronal count at 3, 7 and 14d after CA, was calculated on coronal sections stained with cresyl violet staining. The regions of interest overlap the recorded MRI-ADC hypointense regions and were defined as depicted in in figure 1. They included the primary and secondary motor cortex, the medial ventral and lateral orbital cortex, the frontal cortex (AP 4.68); the caudate putamen (Cpu) (AP 0.60); the CA1, CA2, CA3 field of hippocampus, the dentate girus and hilus (AP-3.48) and were selected manually by a trained expert following Paxinos atlas (Paxinos G, Watson C. The rat brain in stereotaxic coordinates. New York, NY: Academic Press; 2005).

Images in the right and left hemisphere were captured. Neuronal cell loss was expressed as density of cells/mm^2 within each side. The cell count of healthy neurons (and immunostaing quantifications) was performed on the entire ROI of right and left hemispheres as depicted figure 1. Data obtained in each slice per brain area were averaged, thus providing a single value for each brain area, and this value was used for statistical analysis. Neuronal count was assessed by segmentating the cells and excluding the round-shaped signal sized below the area threshold of 25 μm^2 that is known to be associated with glial cells as reported previously.

Number of neurons were manually quantified within hilus, CA1 and CA2 hippocampal subfields. For immunostaining quantifications Iba1 and GFAP, all yielding a sharp signal-to-noise ratio, the positive signal was segmentated by applying a gray-level threshold cutting-off the background. Immunostained areas were expressed as positive pixels/total assessed pixels and reported as the percentage of total stained area as previously described.

Fluoro-Jade

Fluoro-Jade (F-J) labelling was used to detect degenerating neurons as previously described [6].

Degenerating neurons were quantified by reckoning the number of the F-J positive neurons in the ROI's (Figure 1). Data obtained in each slice per brain area were averaged, thus providing a single value for each brain area, and this value was used for statistical analysis.

TUNEL staining

To assess the presence of injured cells showing DNA damage, terminal deoxynucleotidyl transferaseYmediated dUTP nick end labeling (TUNEL) staining was performed on 20-μm sections by in situ cell death detection kit (Roche, Mannheim, Germany) according to the manufacturer instructions, as previously described. Apoptotic cells were quantified by reckoning the number of the TUNEL positive cells in the ROI's (Figure 1). Data obtained in each slice per brain area were averaged, thus providing a single value for each brain area, and this value was used for statistical analysis.

Serum neurofilament light assay

At 3,7 and 14 d after sham or CA surgery, rats were euthanized with an intraperitoneal injection of pentobarbital sodium (150 mg/kg). Blood samples were collected from the descending aorta artery in 3K- EDTA tubes.The plasma NfL levels were detected by a commercial nuclear factor kit (Quanterix, Lexington, MA, USA) of single molecular array immunoassay (SIMOA) on an HD-1 analyzer (Quanterix). Briefly, samples were thawed at 25 °C, and vortexed, 10,000 RCF centrifugation was applied for 5 min. The samples were diluted with sample diluent at a ratio of 1:4 and bonded to paramagnetic magnetic beads on the instrument, which were coated with human NfL-specific antibodies. Then the biotinylated anti-NfL detection antibody was conjugated to streptavidin-β-galactosidase complex, and fluorescence detection was performed. Sample concentrations were calculated from a standard curve, fitted using a four-parameter logistic curve.

Statistical analysis

Data are presented as box and whiskers with line at mean and min-to max. Group size was defined pre-*hoc* setting the Neurological Deficit Score (NDS) at 72 hours after CA/CPR as primary outcome. In line with our previous data to detect effect of CA/CPR (power=0.8; alpha=0.05, 1-sided) the *pre hoc* definition of group size resulted in n=8 as number of rats to be used. Since the mean survival rate at 72 hours after CA was 62%, CA/CPR was performed in 13 rats for longitudinal studies. As secondary outcome, a previously unexplored histopathological assessment, 11 rats (CA/CPR performed in 18 rats) were used for additional sub-groups of post-CA long-term timepoints (3d, n=5; 7d, n=6). The lower number of rats was justified by the drop-out rate of our model. Standard software packages GraphPad Prism (GraphPad Software, Inc, San Diego, CA, version 7.0) were used. Differences between groups over time (group-by-time interaction) for continuous variables were tested using two-way analysis of variance for repeated measurements (time points Groups were compared using One-way ANOVA or Two-way ANOVA followed by an appropriate *post hoc* test. p-values lower than 0.05 were considered statistically significant. Datasets with unequal variances were analyzed using Brown-Forsithe and Welch corrected ANOVA in order to satisfy the assumptions required by the models.

The parametric or non-parametric test was selected after a Shapiro-Wilk normality test to assess whether groups met normal distribution. A detailed description of the test used is provided in the figure legends.

Supplemental **table S1** reports resuscitation outcomes and hemodynamic variables. CA was induced in 13 rats, 12 of which were successfully resuscitated. All data are reported as mean ± SD. ROSC, Return of Spontaneous Circulation. PR, post resuscitation.

**Number of rats subjected to CA**	n=13
**Successful resuscitation**, n	12/13
**Total defibrillations delivered**, n	3 ± 2

**Time to ROSC**, s	518 ± 139
**14 days survival**, n	8/12
**Coronary perfusion pressure**, mmHg	
CPR 2 min	28 ± 8
CPR 4 min	26 ± 7
CPR 6 min	22 ± 5
CPR 8 min	20 ± 4
PR 60 min	92 ± 17
PR 120 min	100 ± 19
PR 180 min	99 ± 15
**Heart rate**, beats/min	
Baseline	354 ± 39
PR 1 h	370 ± 23
PR 2 h	381± 35
PR 3 h	389 ± 22
**Right atrial pressure**, mmHg	
Baseline	4 ± 2
PR 1 h	4 ± 1
PR 2 h	4 ± 1
PR 3 h	3 ± 2
**Mean Arterial Pressure**, mmHg	
Baseline	137 ±14
PR 1 h	109 ± 17
PR 2 h	117 ± 15
PR 3 h	110 ± 17

Supplemental **table S1.**

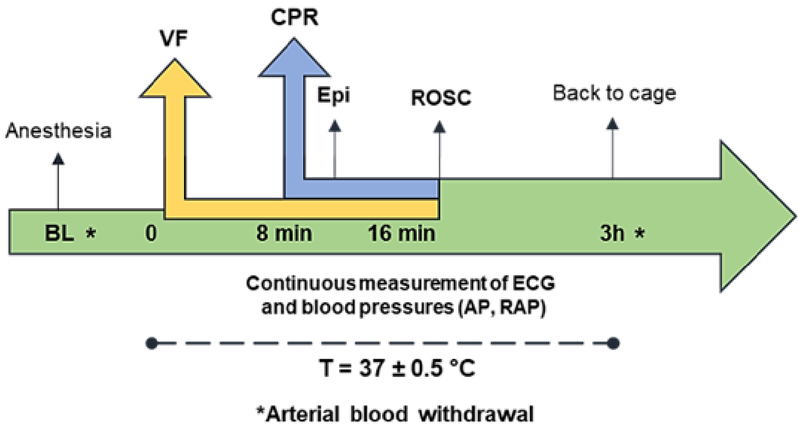

Supplemental figure S2. Experimental model. VF, ventricular fibrillation; CPR, cardiopulmonary resuscitation; Epi, epinephrine; ROSC, return of spontaneous circulation.

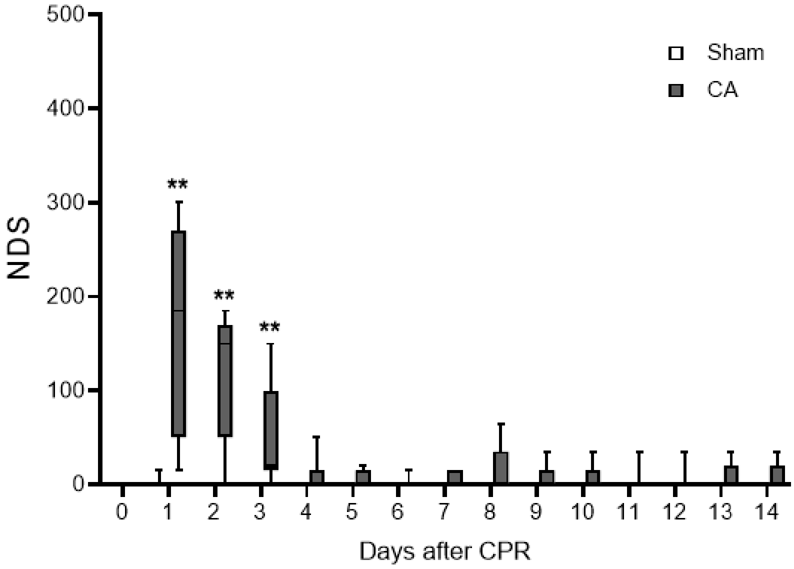

Supplemental figure S3. Behavioral deficits. Neurological deficits (total score 0 to 500) assessed by neurological deficit score (NDS) up to 14 days (d) after CA/CPR[2]. Compared with sham-operated rats CA rats showed sensorimotor dysfunction at 1, 2 and 3d. Two-way ANOVA for repeated measurements followed by Sidak's multiple comparisons test. **$p<0.001$ vs sham. n=8.

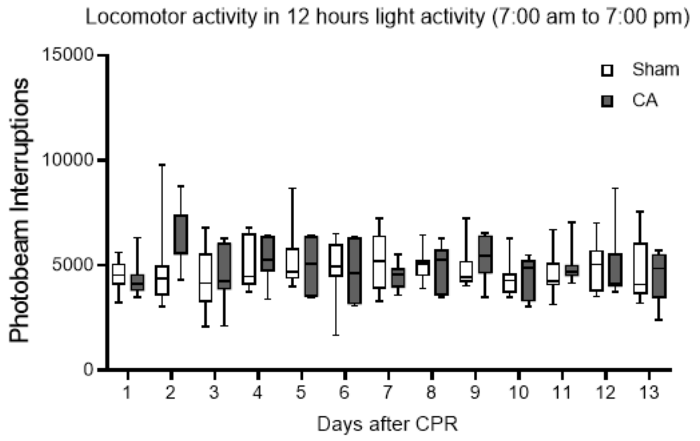

Supplemental figure S4. Locomotor activity in 12 hours light activity. Analysis of locomotor activity in the light phase showed no difference in locomotor activity in CA rats compared to sham animals. Two-way ANOVA for repeated measurements followed by Uncorrected Fisher’s LSD multiple comparisons test. n=8

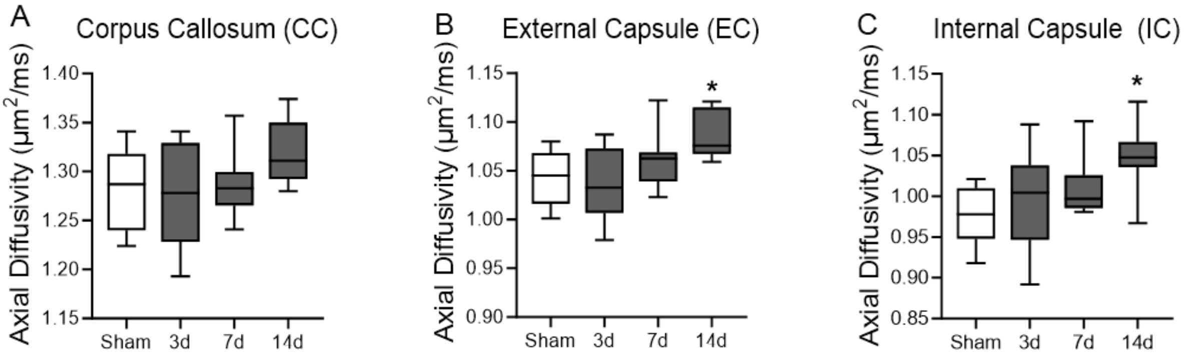

Supplemental figure S5. MRI quantitative analysis of diffusion tensor imaging (DTI-MRI) in the white matter at 3, 7 and 14 d after CA/CPR. Quantification of axial diffusivity (AD) in the corpus callosum (CC, A), external capsule (EC, B) and internal capsule (IC, C). Compared to sham rats, CA induced an increase of AD in the EC (B) and IC (C) at 14d after CPR, with no difference in the CC (A). One-way ANOVA for repeated measurements followed by Tukey's post hoc test. *$p < 0.05$ vs sham. n=8-7-7-8.

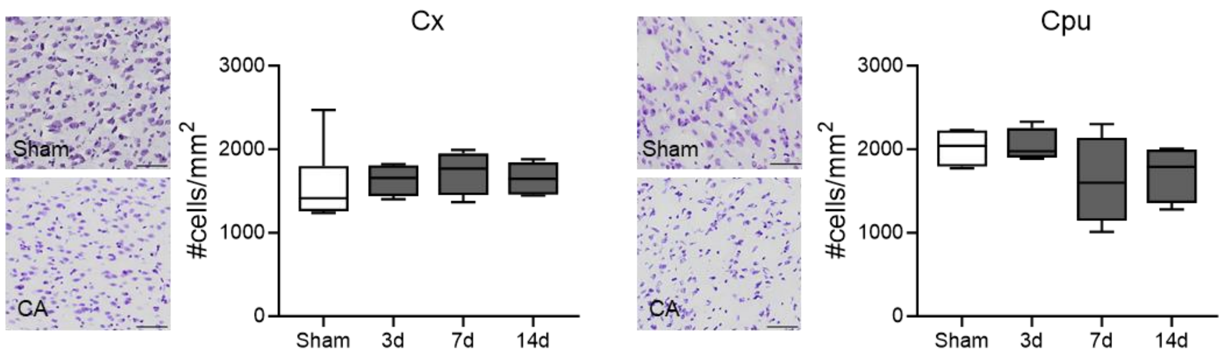

Supplemental figure S6. Representative images of cresyl violet stained neurons in sham and CA rats and quantitative analysis of neuronal density in cortex (A) and Cpu (B). Compared to sham animals, no difference was evidenced in neuronal density of CA rats. One-way ANOVA test. Tukey test for multiple comparisons. n=6-5-6-8. Scale bar, 50 μm.

References

1. Haahr M. Haahr, M. (2024). RANDOM.ORG: True Random Number Service. Available at: https://www.random.org.

2. Neumar RW, Bircher NG, Sim KM, et al. Epinephrine and sodium bicarbonate during CPR following asphyxial cardiac arrest in rats. *Resuscitation* 1995; 29: 249–263.

3. Albertsmeier M, Teschendorf P, Popp E, et al. Evaluation of a tape removal test to assess neurological deficit after cardiac arrest in rats. *Resuscitation* 2007; 74: 552–558.

4. An C, You Y, Park JS, et al. The cut-off value of a qualitative brain diffusion-weighted image (DWI) scoring system to predict poor neurologic outcome in out-of-hospital cardiac arrest (OHCA) patients after target temperature management. *Resuscitation* 2020; 157: 202–210.

5. Wouters A, Scheldeman L, Plessers S, et al. Added Value of Quantitative Apparent Diffusion Coefficient Values for Neuroprognostication After Cardiac Arrest. *Neurology* 2021; 96: e2611–e2618.

6. Laflamme N, Préfontaine P, Rivest S. Fluoro-Jade B Staining for Neuronal Cell Death. *Bio-protocol* 2016; 6: e1702.
